# CEOs’ leadership behaviors and new venture team stability: The effects of knowledge hiding and team collectivism

**DOI:** 10.3389/fpsyg.2022.1001277

**Published:** 2022-11-28

**Authors:** Hongjia Ma, Sisi Tang, Changyi Zhao

**Affiliations:** ^1^School of Business and Management, Jilin University, Changchun, China; ^2^School of Business, Sichuan University, Chengdu, China

**Keywords:** transformational leadership, transactional leadership, knowledge hiding, new venture team stability, team collectivism

## Abstract

**Purpose:**

The reasons for new venture team instability gradually have become a vital issue in the entrepreneurship literature. While chief executive officers’ (CEOs) leadership behaviors is regarded as a critical element of governance within new venture teams, few studies explored the role played by CEOs’ leadership behaviors in new venture team stability. Drawing on the transactional-transformational leadership model, this study divides CEOs’ leadership behaviors in new ventures into two categories, namely, transformational and transactional leadership behaviors. Based on the social exchange theory and the social information processing theory, this study constructs a moderating mediation model to understand how transformational and transactional leadership affects new venture team stability. In this model, knowledge hiding is used as mediating role and team collectivism is used as moderating role.

**Design/methodology/approach:**

Three-wave and two-source data was collected from 66 new ventures in China and an ordinary least squares hierarchical regression model and Hayes’ moderated-mediation approach were applied to test the hypotheses.Findings-The results show transformational leadership and transactional leadership are positively related to new venture team stability. Knowledge hiding mediates the association between transformational leadership and new venture team stability and that between transactional leadership and new venture team stability. Moreover, a high level of team collectivism corresponds to a stronger relationship between transformational leadership and knowledge hiding and a greater indirect effect of transformational leadership on new venture team stability through knowledge hiding.

**Originality/value:**

This study explores the mechanisms and boundary conditions of the effect of transformational leadership, transactional leadership, and new venture team stability, which is an enrichment to the study of governance within new venture teams. It enlightens managers to take effective measures to reduce knowledge hiding and maintain team stability in new venture teams.

## Introduction

Instability of new venture team (NVT instability) emerges as a vital issue in the entrepreneurship literature (Shepherd et al., 2021). NVT are often unstable (Gregori and Parastuty, 2020). Specifically, some team members might doubt whether their teammates are the right people to jointly work with and whether they can push their venture to success, which may trigger conflicts in the team and push some members to leave (Patzelt et al., 2021). Such NVT instability entails serious consequences for the venture. The CB Insights has analyzed 101 failed ventures and found that disharmony among teams is one of the top 20 reasons for failure. Hence, what factors affect NVT instability presents an important research question.

Prior studies have indicated that NVT instability depends on the governance within the team (Slotegraaf and Atuahene-Gima, 2011; Breugst et al., 2015). As a vital element of governance, chief executive officers’ (CEOs) leadership behaviors may affect NVT instability (Shepherd et al., 2021). In particular, CEOs’ leadership behaviors shape individuals’ perceptions, attitudes, and behaviors and strongly influence interpersonal relations, trust, and cooperation among team members (Reid et al., 2018; Yam et al., 2018), all of which are of importance for NVT instability (Gregori and Parastuty, 2020; Lazar et al., 2020). However, few studies explored the role played by CEOs’ leadership behaviors in NVT instability, which reflects a serious research gap.

**Figure 1:**
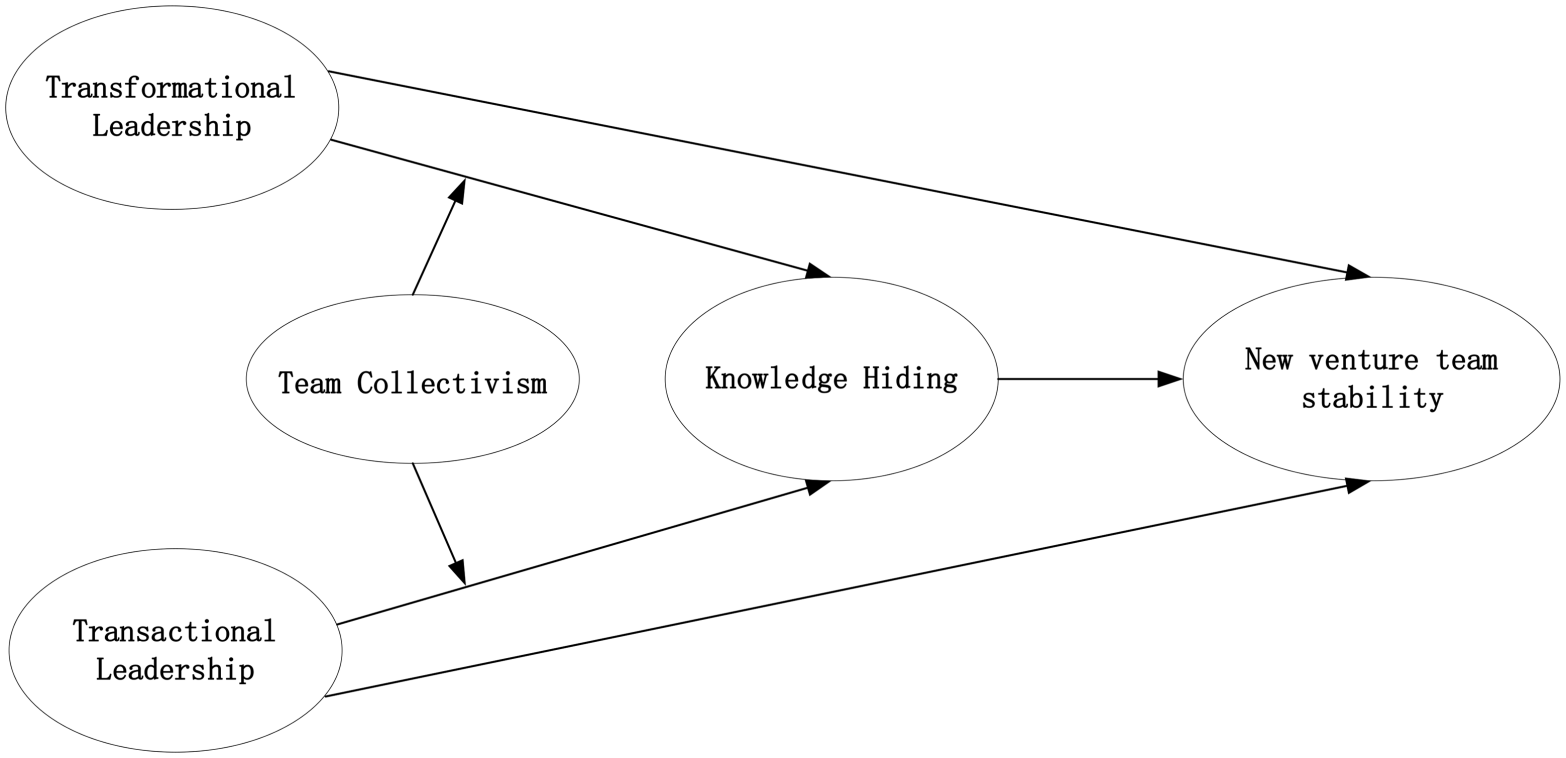
Theoretical model.

We address this gap in three aspects. First, we draw on the transactional-transformational leadership model, which is dominating leadership research. Transformational and transactional are two distinct dimensions of leadership behaviors (Avolio et al., 1999). Researchers on entrepreneurship argued that CEOs’ behaviors in new ventures tend to vary across these two dimensions (Ensley et al., 2006; Kang et al., 2015). Moreover, previous studies suggested that these leadership behaviors are related to interpersonal relations and cooperation among team members (Kovjanic et al., 2013; Gao et al., 2021). Accordingly, this study focuses on testing the impacts of transformational leadership (TFL) and transactional leadership (TAL) on NVT stability. Second, knowledge hiding refers to a deliberate effort on individuals to withhold or conceal important information that coworkers have asked for (Connelly et al., 2012). Recent research in NVTs has highlighted that it is an important process variable that affects team stability (Ma et al., 2022). Based on the social exchange theory, knowledge hiding is regarded as a critical intervention in reciprocal exchange relationships (Wang et al., 2019), which significantly reduces NVT stability (Lee et al., 2017). Moreover, previous research has already corroborated that knowledge hiding is an important underlying influencing mechanism through which leaders reveal their effects on followers’ attitudinal and behavioral outcomes, such as followers’ turnover intentions (Syed et al., 2021). According to the social information processing theory, CEOs’ leadership behaviors can provide cues that inform NVT members about whether knowledge hiding is expected and appropriate behavior (Burmeister et al., 2020). Therefore, this study focuses on knowledge hiding as the important link to examine how TFL and TAL influence NVT stability. Third, collectivism has been identified as a significant moderator in different relationships between leadership and members’ perceptions, attitudes, and behaviors (Walumbwa and Lawler, 2003; Yang et al., 2020). To obtain a more complete understanding of when the association may or may not occur, we take a team collectivism perspective to consider the contextual boundary conditions. As another vital information cue perceived by NVT members, team collectivism influences NVT members’ priority on the needs (Lai et al., 2013), which in turn affects team members’ responses to the CEO’s leadership behaviors (Salancik and Pfeffer, 1978). Therefore, this study examines the moderating effect of team collectivism on the linkage of TFL and TAL to knowledge hiding, and further investigates the moderating role of team collectivism on the indirect effect of TFL and TAL on NVT stability through knowledge hiding. In conclusion, based on the social exchange theory and the social information processing theory, this study constructs a moderating mediation model to explore the influence mechanism between TFL and TAL and NVT stability. The theoretical framework is presented in Figure 1.

This study generates four contributions to the literature. First, our research supplements the emerging field of the governance of NVTs by linking TFL and TAL with team stability through the important mechanism of knowledge hiding in NVTs. Second, this study provides empirical evidence for the positive effects of active leadership on team outcomes in NVTs by examining the impacts of TFL and TAL on knowledge hiding and NVT stability. Third, our study contributes to the knowledge hiding literature by focusing on TFL and TAL as antecedents of knowledge hiding and examining NVT stability as a consequence of knowledge hiding. Last, our study provides a more nuanced understanding of social information processing theory by examining how team collectivism affects team members’ responses to the TFL and TAL behaviors.

## Theoretical background and hypothesis development

### Leadership behaviors and NVT stability

Team stability refers to the extent to which the members of the team remain from beginning to end (Slotegraaf and Atuahene-Gima, 2011). We extend this definition to NVT. NVT is the group of individuals that is chiefly responsible for the strategic decision-making and ongoing operations of a new venture (Klotz et al., 2014). In an NVT, the CEO has central power position and has a more substantial influence on other NVT members and team outcomes (Carmeli et al., 2021). Moreover, this study focuses on what negatively influences NVT members’ perceptions and attitudes and leads them to leave. Hence, we use the term NVT stability to describe the extent to which the members of NVT remain since joining the new venture. If NVT members stay on the team and no changes are made since joining the new venture, then the team is implied fully stable. By contrast, the NVT is considered much less stable if many NVT members exit or some members exit the venture but come back later.

NVT members’ negative attitudes related to interpersonal relations, work, and new venture success leads them to leave (Sun and Wang, 2017; Patzelt et al., 2021). Therefore, TFL and TAL may affect team stability by influencing the perceptions and attitudes of NVT members (Shepherd et al., 2021). Transformational leadership is described by Bass (1985) as a meaningful relationship between the leader and the followers that generates a vision-driven change in followers, goes beyond short-term objectives, and concentrates subordinates’ higher order intrinsic needs (Berraies and Zine El Abidine, 2019). Through the TFL process, NVT members understand the contents and values of entrepreneurial goals, would like to transcend their own interests to pursue collective goals, and thus establish organizational identification (Su et al., 2020). Hekman et al. (2009) argued that team members who have a high level of organizational identification tend to view other team members as “like them” and “on their side.” Therefore, TFL might reduce NVT members’ behaviors that trigger interpersonal disharmony and make team members believe that they are the right people to jointly work and realize entrepreneurial goals. In establishing and realizing collective goals, NVT members can sense that their organization is concerned about their well-being and voices through participative decision-making style and individual consideration practiced by the transformational CEO (Fu et al., 2010; Choi et al., 2016). As a result, they are more committed to their jobs and have higher levels of job satisfaction, reflecting team members’ positive perceptions of work (Choi et al., 2016). Additionally, NVT members will be more optimistic about the new venture’s success by perceiving the CEO’s confidence in their competencies (Nübold et al., 2013). Overall, TFL might enhance NVT stability by reducing team members’ negative attitudes. Hence, we propose the following:

*Hypothesis 1a*: TFL is positively related to NVT stability.

TAL is considered a control-oriented, effective leadership strategy in NVTs (Ensley et al., 2006; Gao et al., 2021). Through the TAL process, each member clears his or her roles, work tasks, and goals (Bass, 1985). Meanwhile, to achieve organizational goals and improve operational efficiency, a transactional CEO tends to establish coordination mechanisms to form some common understanding within the NVT (Gao et al., 2021). As a result, NVT members could experience less conflict with teammates and have a high level of job satisfaction (Chen et al., 2017). Moreover, the effective coordination and explicit roles of team members contribute to making solid decisions in a highly competitive environment and can reduce team members’ anxiety about failure (Hamstra et al., 2014; Hansen and Pihl-Thingvad, 2019; Gao et al., 2021). Additionally, a proactive transactional CEO tends to monitor and rectify any divergence from cooperation in team members’ work (Vaccaro et al., 2012). In this sense, NVT members are more likely to respond to teammates’ requirements and collaborate with teammates, which positively influences team members’ perceptions of interpersonal certainty. Overall, TAL might enhance NVT stability by reducing team members’ negative attitudes about work, venture success, and interpersonal relations. Therefore, we propose the following:

*Hypothesis 1b*: TAL is positively related to NVT stability.

### Mediating role of knowledge hiding

#### Leadership behaviors and knowledge hiding

According to the social information processing theory (Salancik and Pfeffer, 1978), team members’ perceptions, attitudes, and behaviors are shaped by information cues from the social environment. Researchers recently suggested that the CEO can provide cues by his or her leadership behaviors that inform followers about expected and appropriate behaviors (Burmeister et al., 2020), and thus influence knowledge hiding behaviors in the team (Syed et al., 2021; Ma et al., 2022). Based on the transformational–transactional leadership model, the CEO’s TFL and TAL behaviors transfer different information cues to NVT members (excluding the CEO; Kovjanic et al., 2013). A CEO who adopted TFL behaviors would create a common vision in teams, express confidence that goals will be achieved, and inspire team members to transcend their own interests to pursue collective goals (Bass, 1985). In addition, a transformational CEO encourages team members to rethink how they perform their work and provides them with individualized support by developing and coaching each individual in a unique manner (Avolio et al., 1999). Unlike TFL behaviors, a CEO who adopted TAL behaviors would clarify NVT members’ roles, task requirements, and rewards when they complete their work tasks and meet expectations (Bass, 1985). TAL behaviors also include that the CEO monitors team members’ process of completing tasks and solves problems if team members encounter mistakes in completing tasks (Avolio et al., 1999). Therefore, NVT members (excluding the CEO) obtain information about goals, tasks, and rewards in the NVT by observing the CEO’s TFL and TAL behaviors. Through further processing these information, they could know whether knowledge hiding is an expected and appropriate behavior and adjust their behaviors accordingly.

By processing the CEO’s TFL behaviors, NVT members (excluding the CEO) realize that collective goals are emphasized and that individuals are encouraged to enact and realize collective goals with teammates (Choi et al., 2016). As a result, team members tend to think of other teams as rivals and their teammates as allies (Zhu et al., 2019). To deal with complex and creative tasks, they should integrate knowledge by cooperating with allies (Ma et al., 2022). In addition, NVT members (excluding the CEO) are motivated to adopt new methods to complete assignments (Bass, 1985). In this case, team members think that they should seek different views and express new ideas in the NVT (Detert and Burris, 2007; Gao et al., 2021). Integrating knowledge with teammates and emphasizing openness to opinions imply that knowledge hiding is not expected behavior, leading to less knowledge hiding. Therefore, we propose the following:

*Hypothesis 2a*: TFL is negatively related to knowledge hiding among members of NVTs.

Given that valuable knowledge transfer is important for the new venture to gain competitive advantages (Ma et al., 2022), a transactional CEO is more likely to devise incentive systems that recognize individuals’ efforts to create or share knowledge (Du et al., 2013). In this case, getting rewards for knowledge sharing can let NVT members (excluding the CEO) understand that knowledge hiding is not expected behavior in the NVT (Abubakar et al., 2019). In addition, a transactional CEO tends to provide immediate feedback to team members when he or she monitors team members’ processes of completing tasks (Chang et al., 2015). Hence, NVT members (excluding the CEO) might get immediate negative feedback from the CEO when they commit knowledge hiding behavior. Thus, they would think that knowledge hiding is not expected behavior in the workplace. Furthermore, team members know that inappropriate and unethical behaviors are monitored, and thus, they are more likely to reduce the inappropriate knowledge hiding behavior. Therefore, we propose the following:

*Hypothesis 2b*: TAL is negatively related to knowledge hiding among members of NVTs.

#### Knowledge hiding and NVT stability

Social exchange theory posits that members develop relationships based on transactional experience when communicating with coworkers, and they look forward to establishing reciprocal exchange relationships (Blau, 1964). Suppose all members abide by reciprocal norms and have a sense that all exchanges will reach a fair equilibrium over time. In that case, high-quality relationships will be generated among members and evolve into trusting and mutual commitments (Cropanzano and Mitchell, 2005). However, team members assume that those who do not comply are punished, and they adapt their attitudes and behaviors when interventions in the reciprocal exchange process occur (Abubakar et al., 2019). Previous research found that unbalanced social exchange relationships negatively influence individuals’ psychological well-being (Jiang Z. et al., 2019), which may compel them to leave their organizations (Lee et al., 2017).

In recent years, unethical knowledge hiding behavior is regarded as a critical intervention in the reciprocal exchange process and results in unbalanced social exchange relationships between team members (Serenko and Bontis, 2016; Wang et al., 2019). Accordingly, knowledge hiding might exert an important influence on NVT stability. In an NVT context, if knowledge seekers perceive knowledge hiding by teammates, then they may also be reluctant to cooperate and share knowledge with them in the future and even impose social sanctions on them (Zhu et al., 2019). This event results in a reciprocal distrust loop between knowledge seekers and hiders that might undermine good relationships and shared cognition among teammates (Cropanzano and Mitchell, 2005). This reciprocal distrust loop results in team members’ lower perceptions of NVT viability, thereby reducing NVT stability (Klotz et al., 2014; Chen et al., 2017). Moreover, when knowledge hiders experience conflict with reciprocal norms within the NVT and realize that they may be punished as a result, they may experience tension, strain, and reduced job satisfaction, which could encourage them to leave the team (Offergelt et al., 2019). Overall, knowledge hiding might reduce NVT stability by negatively influencing team members’ attitudes related to interpersonal relations, new venture success, and work. Therefore, we propose the following:

*Hypothesis 3*: Knowledge hiding is negatively related to NVT stability.

As stated above, we propose that leadership behaviors influence knowledge hiding behavior among NVT members (excluding the CEO), thereby affecting team stability. The model we develop in Hypotheses 1a, 2a, and 3 allows for the prediction of an indirect relationship between TFL and NVT stability. Specifically, we propose that TFL reduces knowledge hiding by providing cues that inform team members about expected and appropriate behaviors. This case in turn positively influences team members’ attitudes related to interpersonal relations, new venture success, and work and ultimately leads NVT members to stay. Our developed model in Hypotheses 1b, 2b, and 3 leads us to expect an indirect association between TAL and NVT stability. Specifically, we propose that TAL reduces knowledge hiding by providing cues that inform team members about expected and appropriate behaviors and supervision from the CEO, thereby maintaining stability. In sum, we offer the following hypotheses:

*Hypothesis 4a*: Knowledge hiding mediates the relationship between TFL and NVT stability.

*Hypothesis 4b*: Knowledge hiding mediates the relationship between TAL and NVT stability.

### Moderating effect of team collectivism

In addition to expected and appropriate behaviors, NVT members also know which needs could be satisfied while they are doing the expected behaviors by processing the CEO’s TFL and TAL behaviors (Kovjanic et al., 2013). Individuals are more likely to do the expected behaviors when their prioritized needs could be satisfied in the workplace (Salancik and Pfeffer, 1978). Hence, NVT members’ responses to the CEO’s leadership behaviors depend on whether their prioritized needs can be satisfied.

A team is likely to be infiltrated by a collectivist culture when team members stress teamwork in performing team activities and making collective decisions (He et al., 2014). Therefore, NVT members tend to place more priority on the need for relatedness (feeling connected and significant to others) when team collectivism is high (Lai et al., 2013). If team members’ need for relatedness can be satisfied in the NVT, then team members in a high level of team collectivism are more likely to implement the expected behaviors (Salancik and Pfeffer, 1978). From this perspective, team collectivism might affect the effectiveness of leadership behavior and thus influence the relationship between TFL and TAL and knowledge hiding.

A transformational CEO encourages NVT members to enact and realize collective goals with others (Choi et al., 2016) but does not clarify each member’s role and task. By processing these cues, team members understand that cooperation with teammates is important to accomplish creative tasks (Kovjanic et al., 2013; Burmeister et al., 2020), thereby thinking that the need for relatedness can be satisfied when they implement the expected behaviors in the NVT. As a result, we propose that team collectivism may increase the effectiveness of TFL, which strengthens the negative influence of TFL on knowledge hiding. Hence, we suggest the following:

*Hypothesis 5a*: Team collectivism moderates the negative relationship between TFL and knowledge hiding in that the relationship is stronger when team collectivism is high rather than low.

From another aspect, a transactional CEO tends to provide material rewards for team members who implement expected behaviors and complete tasks (Hansen and Pihl-Thingvad, 2019). Thus, NVT members in a high level of team collectivism might not enjoy reducing the inappropriate knowledge hiding because of the gap between the need for material and relatedness. In addition, TAL emphasizes that individuals get rewards for their own performance so that individuals focus on their own tasks and ignore their teammates’ needs in the NVT (Gao et al., 2021). In this case, team members would think that they are not connected with teammates and cannot get support when in trouble. Hence, NVT members would know that their need for relatedness cannot be satisfied in the NVT by processing the CEO’s TAL behaviors, which negatively influences individuals’ motivation to implement the expected behaviors (Deci and Ryan, 2000). Accordingly, we propose that team collectivism may reduce the effectiveness of TAL, which weakens the negative influence of TAL on knowledge hiding. Therefore, we propose the following:

*Hypothesis 5b*: Team collectivism moderates the negative relationship between TAL and knowledge hiding in that the relationship is weaker when team collectivism is high rather than low.

Earlier, we proposed indirect associations between TFL and TAL and NVT stability *via* knowledge hiding (Hypothesis 4a and 4b). Integrating our theorization for the moderating role of team collectivism in the TFL and TAL–knowledge hiding association, team collectivism will likely influence the strength of the indirect relationship between TFL and TAL and NVT stability through knowledge hiding conditionally (Edwards and Lambert, 2007; Wang et al., 2016). When team collectivism is high rather than low, TFL has a stronger effect on NVT stability because NVT members enjoy reducing knowledge hiding behaviors by processing the CEO’s leadership behaviors; TAL has a weaker effect on NVT stability because NVT members have insufficient incentives to reduce knowledge hiding behaviors by processing the CEO’s leadership behaviors. This notion indicates a moderated mediation model of the relationship between the study’s variables, as depicted in Figure 1. Thus, we set forth the following hypotheses:

*Hypothesis 6a*: Team collectivism moderates the indirect effect of TFL on NVT stability through knowledge hiding in that the indirect relationship is stronger when team collectivism is high rather than low.

*Hypothesis 6b*: Team collectivism moderates the indirect effect of TAL on NVT stability through knowledge hiding in that the indirect relationship is weaker when team collectivism is high rather than low.

The theoretical model of this study is shown in Figure 1.

## Research methods

### Sample

Zahra and Bogner (2000) defined new venture as a company that is 8 years old and under. Therefore, we selected NVTs from companies younger than 8 years of age. We collected data from 66 new ventures in the Jilin province of China (45 manufacturing enterprises and 21 non-manufacturing companies). We approached members of NVTs engaged with a research project in cooperation with Jilin Provincial Science and Technology Department. In the final sample, team size ranged from four to eight members (Mean = 5.79, Standard Deviation (SD) = 0.95). Among the 382 member respondents, 31.6% joined or started a business, 63.9% were male, and 86.3% had a bachelor’s degree or higher. The average age of the members was 35.4 years of age (SD = 6.77), and the average age of the CEOs who underwent individual interviews was 40.14 years (SD = 4.9), of which 25.8% were female.

### Procedures

A cross-sectional study may introduce deviations, such as consistency motifs and illusionary correlations; thus, a three-stage longitudinal research design was adopted. However, one cannot take advantage of such a research design if the time lag is too short or too long as it is not conducive to studying the accuracy of the relationship between the measured variables (Peng, 2013). Thus, the present study used a two-month lag to compensate for this fact. As this study adopted team-level data to analyze the relationship between the constructs, we scheduled meetings with the CEOs of 98 new ventures who agreed to participate before our survey. In these meetings, we explained the purpose of the survey, made a commitment to information security for the respondents, and asked for their help in identifying NVT members, distributing the questionnaires at their firms, and then collecting the data. In the first stage (Time 1), the NVT members (excluding CEOs) completed the survey about leadership behaviors. A total of 585 members belonging to 98 teams returned the questionnaires. Two months later (Time 2), we asked all 585 respondents to accomplish a second questionnaire on team collectivism, knowledge hiding, and demographic information. In Time 2, 480 members belonging to 82 teams completed the second-stage survey, representing an 82.05% response rate. Approximately another 2 months later (Time 3), the CEOs of the 82 teams (one per team) were asked to answer a third-stage questionnaire about the basic situation of the firm and team stability; 74 CEOs returned the questionnaires (90.24% response rate). To increase data quality and reliability, we also illustrated the purpose of this study and assured anonymity and confidentiality by a cover letter attached to the questionnaires. Each survey featured an anonymous code so that we could identify the three waves of the questionnaires. After removing incomplete and unmatched surveys, a final matched sample of 382 team members nested within 66 NVTs was obtained.

### Measures

Given that the respondents are from China, we used a back-translation procedure (Brislin, 1986) for the English questionnaire, which we translated into Chinese and then retranslated into English for comparison to ensure accuracy. We used measuring instruments from the extant literature and a Likert-type response scale for all items (Appendix A) ranging from 1 (“strongly disagree”) to 5 (“strongly agree”). As shown in the following section, four variables (i.e., TFL, TAL, team collectivism, and knowledge hiding) measured at the individual level have acceptable consistency and reliability and are aggregated on the team level.

#### Leadership behaviors

The study adopted Avolio et al. (1999) measures for TFL and TAL. We used a 20-item scale to measure TFL (12 items on charisma and inspirational leadership, four items on intellectual stimulation, and the remaining four items on individualized consideration). Examples of sample items include “On our team, the CEO goes beyond self-interest for the good of the group” and “On our team, the CEO re-examines critical assumptions.” The resulting Cronbach’s alpha value confirmed the good internal consistency and reliability of the scale (alpha = 0.90). The intraclass correlations (ICCs) were also computed to evaluate agreement between team members; ICC(1) was 0.18, and ICC(2) was 0.56. The mean and median r_wgs_ were 0.96 and 0.97, respectively. In addition, we used an eight-item scale to measure TAL, where four of which are for contingent rewards, and the remaining four are for management by exception. Examples of sample items include “On our team, the CEO clarifies what I can expect to receive when goals are achieved” and “On our team, the CEO focuses on my mistakes.” The scale showed a reliability value of 0.84, an ICC(1) of 0.27, and an ICC(2) of 0.67. The mean and median r_wgs_ were 0.93 and 0.95, respectively.

#### Team collectivism

We adopted He et al. (2014) seven-item scale to measure team collectivism, with sample items, such as “The CEO is protective of and generous to loyal workers.” The scale had a reliability of 0.94, an ICC(1) value of 0.26, and an ICC(2) value of 0.66. The mean and median r_wgs_ were 0.92 and 0.94, respectively.

#### Knowledge hiding

We used 12 items adapted from Connelly et al. (2012) to measure knowledge hiding (including playing dumb, evasive hiding, and rationalized hiding). Examples of sample items include “When my teammates asked for some information, I pretended that I did not know the information” and “When my teammates asked for some information, I agreed to help them but never really intended to.” The scale showed a reliability value of 0.83, an ICC (1) value of 0.39, and an ICC(2) value of 0.78. The mean and median r_wgs_ were 0.92 and 0.94, respectively.

#### NVT stability

We measured NVT stability using three items adapted from the team stability scale of Slotegraaf and Atuahene-Gima (2011). An example of a sample item is “NVT members (excluding the CEO) remained since joining the new venture.” The Cronbach’s alpha value was 0.88.

#### Control variables

This study controlled for firm age (years since founding) and team size and industry variables that have been selected as factors influencing team stability in previous research (Ucbasaran et al., 2003). We coded team size as a continuous variable and industry as a dummy variable (1 = manufacturing, 0 = non-manufacturing). Considering that the age of a new venture is 8 years old and under, we classified all new ventures according to firm age; a firm age of less than 2, 2–4, 4–6, and 6–8 years, thereby controlling for venture firm age.

## Results

### Analysis

Exploratory factor analysis on TFL, TAL, team collectivism, knowledge hiding, and NVT stability was conducted to examine the discriminant validity of the measures. Table 1 shows the analysis results. Factor analysis using principal component extraction with orthogonal rotation extracted nine clear factors with eigenvalues greater than 1.0. Two types of CEOs’ TAL behaviors were extracted as a factor. The factor loading coefficient of the item on the corresponding factor exceeded 0.40 and was thus significant (Hair et al., 1998). As Cronbach’s alpha values were > 0.70, the resulting scales have good reliability (Hair et al., 1998).

**Table 1:** Factor analysis.

Items	CH	IS	IC	TAL	TC	PD	EH	RH	NVTS
Transformational leadership (TFL)									
CH1	*0.79*	0.01	−0.09	0.08	0.08	−0.18	0.03	−0.11	0.1
CH2	*0.80*	0.21	−0.07	−0.00	0.13	−0.06	−0.19	−0.07	0.08
CH3	*0.75*	0.26	0.01	−0.09	−0.04	−0.01	0.06	−0.21	0.11
CH4	*0.75*	0.07	0.20	−0.03	0.03	−0.06	−0.10	0.04	−0.02
CH5	*0.73*	0.2	0.19	0.10	0.12	−0.17	−0.05	0.08	0.12
CH6	*0.72*	−0.12	0.13	0.26	0.13	−0.08	−0.14	0.06	−0.03
CH7	*0.69*	0.16	0.02	0.06	0.08	0.02	−0.01	−0.42	0.13
CH8	*0.85*	0.21	0.07	0.04	0.10	−0.15	−0.22	−0.04	0.15
CH9	*0.81*	0.23	−0.12	0.03	0.11	−0.1	−0.08	−0.20	0.12
CH10	*0.85*	0.04	0.09	0.16	0.11	−0.16	−0.11	−0.22	0.10
CH11	*0.85*	0.11	0.21	0.11	0.11	−0.14	−0.21	−0.07	0.06
CH12	*0.88*	0.10	0.09	0.16	0.08	−0.11	−0.08	−0.22	0.12
IS1	0.44	*0.75*	−0.03	0.12	−0.01	−0.01	−0.11	−0.14	−0.06
IS2	0.06	*0.81*	0.06	0.07	0.05	−0.11	−0.15	−0.14	−0.02
IS3	0.39	*0.87*	0.07	0.05	0.03	−0.13	−0.06	−0.10	−0.04
IS4	0.32	*0.75*	0.18	0.02	0.10	−0.23	0.01	−0.00	0.15
IC1	0.08	0.13	*0.83*	−0.05	0.19	−0.07	0.04	−0.07	−0.02
IC2	0.15	0.04	*0.87*	−0.07	0.10	−0.02	0.01	−0.06	0.07
IC3	0.17	0.03	*0.83*	0.02	0.15	−0.04	0.03	−0.08	0.03
IC4	0.02	0.02	*0.85*	0.01	0.11	0.01	−0.10	−0.13	−0.01
Transactional leadership (TAL)									
CR1	0.03	0.04	0.07	*0.90*	0.06	−0.05	0.02	−0.06	0.07
CR2	−0.07	0.19	−0.09	*0.82*	−0.05	0.05	0.08	0	0.1
CR3	0.04	0.03	−0.12	*0.78*	0.02	−0.11	0.01	0.17	0.17
CR4	−0.01	0.08	−0.03	*0.88*	−0.03	−0.08	0.05	0.12	0.20
ME1	0.30	−0.07	0.04	*0.75*	−0.01	−0.09	−0.19	−0.03	−0.04
ME2	0.02	0.12	0.10	*0.81*	0.00	−0.09	−0.28	−0.16	0.03
ME3	0.30	−0.13	−0.13	*0.69*	0.16	−0.07	−0.23	−0.11	0.16
ME4	0.24	−0.05	0.02	*0.82*	0.07	−0.08	−0.14	−0.13	0.03
Team collectivism									
TC1	0.09	−0.06	0.14	0.04	*0.92*	−0.09	−0.04	−0.03	−0.03
TC2	0.04	0.02	0.14	0.07	*0.91*	−0.08	−0.07	0.02	0.08
TC3	0.14	−0.01	0.07	0.07	*0.89*	−0.14	−0.09	−0.02	−0.03
TC4	0.05	−0.02	0.02	0.03	*0.94*	−0.16	0.04	−0.04	0.07
TC5	0.11	0.03	0.04	−0.06	*0.94*	−0.06	−0.06	−0.05	−0.01
TC6	0.13	0.08	0.12	−0.00	*0.90*	−0.06	−0.06	−0.02	0.02
TC7	0.08	0.12	0.07	−0.01	*0.93*	−0.03	0.02	−0.02	0.00
Knowledge hiding									
PD1	−0.08	−0.17	−0.08	−0.07	−0.18	*0.80*	0.12	0.32	−0.08
PD2	−0.30	−0.01	−0.03	−0.12	−0.08	*0.80*	0.17	0.16	−0.15
PD3	−0.21	−0.16	0.04	−0.11	−0.22	*0.78*	0.16	0.19	−0.01
PD4	−0.21	−0.11	−0.09	−0.12	−0.21	*0.84*	0.17	0.27	−0.15
EH1	−0.14	−0.17	0.01	−0.17	−0.19	0.33	*0.49*	0.33	−0.24
EH2	−0.37	−0.05	−0.04	−0.23	0.02	0.19	*0.66*	0.13	−0.25
EH3	−0.29	−0.17	−0.02	−0.11	−0.13	0.23	*0.79*	0.12	−0.16
EH4	−0.29	−0.13	0.03	−0.10	−0.15	0.38	*0.63*	0.14	−0.36
RH1	−0.11	−0.12	−0.11	0.02	−0.04	0.39	0.06	*0.80*	−0.08
RH2	−0.11	0.03	−0.19	0.01	−0.00	0.24	0.02	*0.77*	−0.23
RH3	−0.31	−0.1	−0.05	−0.14	0.07	0.09	0.17	*0.77*	0.08
RH4	−0.17	−0.18	−0.08	0.01	−0.14	0.22	0.13	*0.78*	−0.09
New venture team (NVT) stability									
ETS1	0.19	0.07	−0.02	0.20	0.02	0.00	−0.27	−0.16	*0.78*
ETS2	0.13	−0.07	0.01	0.23	0.01	−0.16	−0.06	−0.09	*0.82*
ETS3	0.27	0.03	0.10	0.15	0.02	−0.18	−0.19	−0.07	*0.83*

CH, charisma and inspirational leadership; IS, intellectual stimulation; IC, individualized consideration; CR, contingent rewards; ME, management by exception; TC, team collectivism; PD, playing dumb; EH, evasive hiding; RH, rationalized hiding; NVTS, new venture team stability; TAL, transactional leadership; The values in italics are loadings for each item that are above the recommended value of 0.4.

To test these hypotheses, we employed and examined a series of hierarchical regression models (1) to (4), using SPSS 20.0.

Model (1) was constructed based on H1a and H1b to detect the impact of TFL and TAL on NVT stability. If the main coefficient β_1_ of TFL and β_2_ of TAL are significantly positive, it would confirm that TFL and TAL can improve NVT stability.


(1) $$\begin{array}{l} NVT\;{\mathrm{stability}}_{i}=\beta_{0}+\beta_{1}\;{\mathrm{TFL}}_{i}+\beta_{2}\;{\mathrm{TAL}}_{i}\\ +\beta_{3}\;\mathrm{Firm}\;\mathrm{age}+\beta_{4}\;\mathrm{Industry}+\beta_{5}\;\mathrm{Team}\;{\mathrm{size}}_{i}+\varepsilon_{i} \end{array}$$

Model (2) was constructed based on H2a and H2b to detect the impact of TFL and TAL on knowledge hiding. If the main coefficient β_1_ of TFL and β_2_ of TAL are significantly negative, it would confirm that TFL and TAL can reduce knowledge hiding.


(2) $$\begin{array}{l} \mathrm{Knowledge}\;{\mathrm{hiding}}_{i}=\beta_{0}+\beta_{1}\;{\mathrm{TFL}}_{i}+\beta_{2}\;{\mathrm{TAL}}_{i}\\ +\beta_{3}\;\mathrm{Firm}\;\mathrm{age}+\beta_{4}\;\mathrm{Industry}+\beta_{5}\;\mathrm{Team}\;{\mathrm{size}}_{i}+\varepsilon_{i} \end{array}$$

Model (3) was constructed based on H3 to detect the impact of knowledge hiding on NVT stability. If the main coefficient β_1_ of knowledge hiding is significantly negative, it would confirm that knowledge hiding can reduce NVT stability


(3) $$\begin{array}{l} NVT\;{\mathrm{stability}}_{i}=\beta_{0}+\beta_{1}\;\mathrm{Knowledge}\;{\mathrm{hiding}}_{i}\\ +\beta_{2}\;\mathrm{Firm}\;\mathrm{age}+\beta_{3}\;\mathrm{Industry}+\beta_{4}\;\mathrm{Team}\;{\mathrm{size}}_{i}+\varepsilon_{i} \end{array}$$

To test the moderating effect of team collectivism proposed by H5a and H5b, we introduced the intersections term TFL× team collectivism and TAL× team collectivism in the model (4). The coefficient β_3_ and β_4_ in the model (4) was expected to be significantly negative if H5a and H5b were confirmed.


(4) $$\begin{array}{l} \mathrm{Knowledge}\;{\mathrm{hiding}}_{i}=\beta_{0}+\beta_{1}\;{\mathrm{TFL}}_{i}+\beta_{2}\;{\mathrm{TFL}}_{i}\\ +\beta_{3}\;{\mathrm{TFL}}_{i}\times \mathrm{Team}\;{\mathrm{collectivism}}_{i}+\beta_{4}\;{\mathrm{TAL}}_{i}\\ \times \mathrm{Team}\;{\mathrm{collectivism}}_{i}+\beta_{5}\;\mathrm{Team}\;{\mathrm{collectivism}}_{i}\\ +\beta_{6}\;\mathrm{Firm}\;{\mathrm{age}}_{i}+\beta_{7}\;{\mathrm{Industry}}_{i}+\beta_{8}\;{\mathrm{Teamsize}}_{i}+\varepsilon_{i} \end{array}$$

Where, i = firm; ɛ_i_ is the observation error.

Considering the advantages of the bootstrapping method, Hayes (2009) bootstrapping-based (moderated) mediation analysis was adopted to assess the mediating effect of knowledge hiding. Moreover, we performed the bootstrapping method of Preacher et al. (2007) to test the moderated mediation.

### Hypotheses testing

Table 2 presents the descriptive statistics and correlations for all of the variables. TFL was positively related to team collectivism (*r* = 0.27, *p* < 0.05) and NVT stability (*r* = 0.38, *p* < 0.01) and negatively related to knowledge hiding (*r* = −0.56, *p* < 0.01). TAL was positively related to NVT stability (*r* = 0.35, *p* < 0.01) and negatively related to knowledge hiding (*r* = −0.29, *p* < 0.05). Knowledge hiding was negatively related to NVT stability (*r* = −0.51, *p* < 0.01). We used ordinary least squares regression analysis to test the hypotheses. The maximum value of the variance inflation factor from the analyses is 1.278, which is substantially below the general cut-off value of 10. This result suggests that the likelihood that multicollinearity is a problem is minimal.

**Table 2:** Means, standard deviations, correlations, and reliability values.

Variables		Mean	SD	1	2	3	4	5	6	7	8
1	Firm age	3.15	1.11	_							
2	Industry	0.73	0.45	0.21	_						
3	Team size	5.79	1.20	−0.02	0.03	_					
4	TFL	3.75	0.33	−0.04	0.11	0.12	(0.90)				
5	TAL	3.43	0.36	0.13	0.12	0.03	0.22	(0.84)			
6	Team collectivism	3.78	0.51	−0.02	−0.10	−0.02	0.27*	0.07	(0.94)		
7	Knowledge hiding	3.29	0.45	0.23	0.07	−0.08	−0.56**	−0.29*	−0.27*	(0.83)	
8	NVT stability	3.70	0.89	0.13	0.03	0.08	0.38**	0.35**	0.10	−0.51**	(0.88)

N = 66; Reliability is shown on the diagonal within parentheses; TFL, transformational leadership; TAL, transactional leadership; NVT stability, new venture team stability; SD: standard deviation; *p < 0.05; **p < 0.01.

Hypothesis 1a predicts a positive relationship between TFL and NVT stability, which is confirmed by the regression analysis results in Table 3 (model 5; for TFL, *β* = 0.33, *p* < 0.01). Meanwhile, Hypothesis 1b proposes that TAL would be positively related to NVT stability, which is also confirmed by the results in Table 3 (model 5; for TAL, *β* = 0.27, *p* < 0.05). Specifically, the results show that an increase of one standard deviation in TFL (0.33) and TAL (0.36) will lead to increases in the level of NVT stability of 10.89 percent (0.33 × 0.33) and 9.72 percent (0.36 × 0.27).

**Table 3:** Regression analysis and tests of Hypotheses 1a, 1b, 2a, 2b, 3, 5a, and 5b.

	Knowledge hiding			New venture team stability		
	M1	M2	M3	M4	M5	M6
Firm age	0.22	0.22*	0.25*	0.13	0.12	0.26*
Industry	0.02	0.10	0.03	−0.01	−0.07	0.01
Team size	−0.08	−0.01	−0.03	0.08	0.04	0.04
TFL		−0.52***	−0.40***		0.33**	
TAL		−0.22*	−0.26*		0.27*	
Team collectivism			−0.15			
Knowledge hiding						−0.57***
TFL × Team collectivism			−0.27*			
TAL × Team collectivism			0.03			
Overall model F	1.31	8.49**	7.07**	0.49	3.69**	7.39**
*R* ^2^	0.06	0.41	0.50	0.02	0.24	0.33
Adjusted *R*^2^	0.01	0.37	0.43	−0.02	0.17	0.28

N = 66; Standardized regression coefficients are reported; TFL, transformational leadership; TAL, transactional leadership; *p < 0.05. **p < 0.01; *** *p* < 0.001.

Hypothesis 2a predicts that TFL would be negatively associated with knowledge hiding. The results in Table 3 confirm this relationship (model 2; for TFL, *β* = −0.52, *p* < 0.001), thereby supporting Hypothesis 2a. Similarly, Hypothesis 2b expects a negative relationship between TAL and knowledge hiding. Again, Table 3 validates such an association (model 2; for TAL, *β* = −0.22, *p* < 0.05), thereby supporting Hypothesis 2b. Specifically, the results show that an increase of one standard deviation in TFL (0.33) and TAL (0.36) will lead to decreases in the level of knowledge hiding of 17.16 percent (0.33 × 0.52) and 7.92 percent (0.36 × 0.22).

By comparing TFL and TAL, we further find that the effect of TFL on NVT stability (model 5; for TFL, *β* = 0.33, *p* < 0.01) is greater than TAL (model 5; for TAL, *β* = 0.27, *p* < 0.05). Meanwhile, the effect of TFL on knowledge hiding (model 2; for TFL, *β* = −0.52, *p* < 0.001) is greater than TAL (model 2; for TAL, *β* = −0.22, *p* < 0.05). The results indicate that both TFL and TAL can reduce knowledge hiding and maintain NVT stability, but their degree is different.

Hypothesis 3 predicts a negative association between knowledge hiding and NVT stability. According to Table 3, knowledge hiding negatively affects NVT stability (model 6, *β* = −0.57, *p* < 0.001), supporting Hypothesis 3. Specifically, the result shows that an increase of one standard deviation in knowledge hiding (0.45) will lead to a decrease in the level of NVT stability of 25.26 percent (0.45 × 0.57).Hypothesis 4a and 4b propose that TFL and TAL would indirectly influence NVT stability through knowledge hiding. This study used a bootstrapping-based mediation analysis approach (Hayes, 2009) to test the indirect effect. Using the SPSS 20.0 macro program Process 3.3, we found a significant indirect effect of TFL on NVT stability through knowledge hiding (based on 5,000 iterations at the 95% bootstrap confidence interval CI = [0.37, 1.30], not containing zero). Thus, Hypothesis 4a is supported. The bootstrapping test also indicates a significant indirect effect of TAL on NVT stability through knowledge hiding (based on 5,000 iterations at the 95% bootstrap confidence interval CI = [0.02, 0.85], not containing zero), which, in turn, supports Hypothesis 4b.

**Figure 2:**
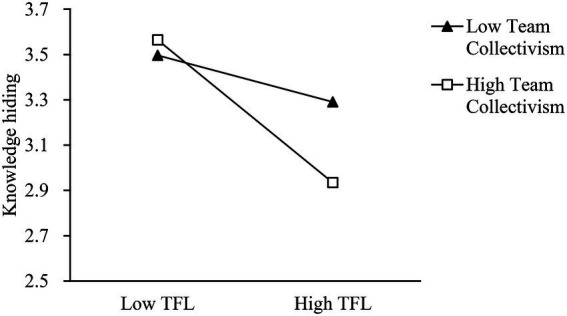
Team collectivism as a moderator of the relationship between TFL and knowledge hiding.

According to Hypothesis 5a, team collectivism moderates the relationship between TFL and knowledge hiding. The results in Table 3 indicate that team collectivism significantly strengthens the effect of TFL on knowledge hiding (model 3; for TFL × TC, *β* = −0.27, *p* < 0.05), thereby supporting Hypothesis 5a. Figure 2 shows this significant interaction (Aiken and West, 1991). The simple slope increases with the increase of the value of moderation variable, and is significantly not 0. Consistent with Hypothesis 5a, the TFL–knowledge hiding relationship is stronger for teams with a high level of collectivism than those with a low level of collectivism. Hypothesis 5b supposes that team collectivism weakens the relationship between TAL and knowledge hiding. However, the results in Table 3 show a non-significant interaction term for TAL and team collectivism (model 3; for TAL × TC, *β* = 0.03, *p* > 0.05), which rejects Hypothesis 5b. The results indicate that team collectivism cannot strengthen or weaken the association between TAL and knowledge hiding.

Hypothesis 6a and 6b propose that team collectivism would moderate the indirect effects of TFL and TAL on NVT stability through knowledge hiding. Table 4 represents the conditional indirect effects of TFL on NVT stability through knowledge hiding across different levels (i.e., mean − 1 SD, mean, and mean + 1 SD) of team collectivism. The results show that the conditional indirect effect of TFL significantly strengthened when the degree of team collectivism is higher (low = 0.12, non-significant; medium = 0.25, significant; high = 0.34, significant). Therefore, team collectivism also reinforces the indirect effect of TFL on NVT stability through knowledge hiding. Hypothesis 6a is supported. The precondition for Hypothesis 6b is dismissed because of Hypothesis 5b, and Hypothesis 6b is also rejected.

**Table 4:** Conditional indirect effect testing for Hypothesis 6a.

Level	TC	Conditional Indirect effect	BootSE	LLCI 95%	ULCI 95%
Low = mean – 1SD	−0.97	0.12	0.10	−0.09	0.32
Medium = mean	0.13	0.25	0.09	0.10	0.43
High = mean + 1SD	0.88	0.34	0.10	0.15	0.55

N = 66; Bootstrap sample size = 5,000; 95% bias-corrected confidence intervals are reported; TC, team collectivism; SE, standard error; LLCI, lower limit confidence interval; ULCI, upper limit confidence interval.

## Discussion

As noted, stability is important for NVTs, but investigation of the antecedents of NVT stability is limited, specifically from the leadership behavior perspective. However, CEOs’ leadership behaviors have significant impacts on the operation of new ventures (Reid et al., 2018). To bridge the gap, this study examines whether, how, and when TFL and TAL influence NVT stability by integrating social exchange theory and social information processing theory. Our empirical results suggest that TFL and TAL are facilitators of NVT stability and are inhibitors of knowledge hiding among NVT members (excluding the CEO), but TFL has effects on NVT stability and knowledge hiding beyond the effects of TAL. Moreover, TFL and TAL can improve NVT stability by reducing knowledge hiding. When team collectivism is high rather than low, TFL has a stronger indirect effect on NVT stability through knowledge hiding. However, team collectivism cannot moderate the negative association between TAL and knowledge hiding. Some team collectivism traits may explain these phenomena. On the one hand, as previously mentioned, team members with a high level of team collectivism know that their prioritized needs cannot be satisfied in the NVT by processing the CEO’s TAL behaviors, which may not reduce inappropriate knowledge hiding. On the other hand, Jiang et al. (2016) argued that conflict avoidance, compromise, and endurance are deeply rooted in highly collectivistic cultures. Therefore, NVT members in a high level of team collectivism tend to do expected behaviors to avoid conflict and maintain interpersonal harmony. As a result, members’ preference of conflict avoidance, compromise, and endurance may mitigate the effects caused by the unsatisfied need.

### Theoretical contributions

This study makes four sets of unique contributions. First, this study extends the literature on the governance of NVTs from social exchange theory and social information processing theory by estimating how TFL and TAL stimulate NVT stability. The extant literature on the antecedents of NVT stability focused on individuals’ feelings of loss in power over the direction of the venture (Shepherd et al., 2021), interpersonal relationships and conflicts at the team level, and investors’ influence at the organizational level (Gregori and Parastuty, 2020). Research on how antecedents related to leadership influence NVT stability is limited but highly important. Therefore, our finding enriches existing literature about NVT stability by revealing the positive effects of TFL and TAL on NVT stability. In addition, our empirical results indicate that knowledge hiding is an important bridge between TFL and TAL and NVT stability. Thus, this study also introduces the underlying mechanism for understanding the relationship between CEOs’ leadership behaviors and NVT stability.

Second, this study provides empirical evidence for the positive effects of active leadership on team outcomes in NVTs by examining the impacts of TFL and TAL on NVT stability and knowledge hiding. TFL and TAL are regarded as active leadership, but they have different influences on outcomes (Ryan and Tipu, 2013). Previous studies have consistently supported the positive relationships between TFL and outcomes such as knowledge management process, performance, satisfaction, engagement, and turnover (Birasnav, 2014; Siangchokyoo et al., 2020), but have revealed mixed results with regard to the relationships between TAL and these outcomes (Jiang L. et al., 2019; Lee et al., 2019; Young et al., 2021). The results from our research demonstrate that active leadership (including TFL and TAL) can reduce knowledge hiding and maintain team stability in NVTs and the effects of TFL on NVT stability and knowledge hiding beyond the effects of TAL. Thus, this study extends the literature on the effects of active leadership in the NVT context.

Third, this study explores the antecedents and consequences of knowledge hiding by examining the impacts of TFL and TAL on NVT stability. According to social exchange theory (Blau, 1964), knowledge hiding destroys positive reciprocal exchange relationships and negatively influences individuals’ psychological well-being. Therefore, researchers exerted major effort to identify the consequences of knowledge hiding, such as individuals’ innovative behavior (Guo et al., 2022), individual and team creativity (Wang et al., 2019), and team performance (Zhang and Min, 2019). However, the negative consequences of knowledge hiding in NVTs have been overlooked. The results from our research demonstrate that knowledge hiding negatively affects NVT stability by negatively influencing team members’ attitudes related to interpersonal relations, venture success, and work. Therefore, this study attempts to bridge the gap between knowledge management and NVT governance literature. About the antecedents relevant to CEOs of knowledge hiding, the abusive supervision of leaders (Feng and Wang, 2019), leader-member exchanges (Zhao et al., 2019), and exploitative leadership (Syed et al., 2021) are related to knowledge hiding at mature firms. However, investigation of the relationship between TFL and TAL and knowledge hiding is still limited. From a social information processing perspective, this study regards TFL and TAL as inhibitors of knowledge hiding. The results reveal that TFL and TAL can significantly reduce knowledge hiding in the NVT context. Therefore, this study provides a basic and solid foundation for future studies regarding knowledge hiding.

Last, this study offers a deeper understanding of social information processing theory by combining two important cues in the social environment (i.e., TFL, TAL, and team collectivism) that affect the knowledge hiding behaviors among NVT members (excluding the CEO). Previous studies focused more on the effect of CEOs’ leadership behaviors and team culture on individuals’ behaviors, respectively. However, little is known about how individuals’ behaviors are simultaneously influenced by these two informational cues, particularly for knowledge hiding behaviors of NVT members (Yam et al., 2018; Lei et al., 2021). The study confirms that team collectivism enhances the effectiveness of TFL and reduces knowledge hiding in NVTs. Thus, our study implies that individuals are more likely to implement expected behaviors when the CEO’s leadership behaviors show that NVT members’ prioritized needs shaped by information cues at the team level can be satisfied in the workplace. Hence, this study extends the literature on social information processing theory by exploring the moderating effect of team collectivism on the relationship between TFL and TAL and knowledge hiding in NVTs.

### Managerial implications

This study also offers four practical implications for NVT members. First, the study found that TFL and TAL are positively associated with NVT stability. Therefore, CEOs could improve NVT stability by adopting TFL and TAL behaviors depending on the specific context. Furthermore, the study indicates TFL has an effect on NVT stability beyond the effect of TAL. Hence, TFL could be prioritized by CEOs. Moreover, CEOs could participate in training sessions in which they can reflect on their own leadership behaviors and learn how to effectively implement TFL and TAL behaviors to realize some leadership functions (Burmeister et al., 2020).

Second, given the importance of knowledge hiding for NVT stability, NVT members should pay more attention to the knowledge management process, particularly to knowledge hiding. Peng (2013) suggested that members who have strong territorial feelings regarding their own knowledge are more likely to withhold knowledge. Therefore, NVT members can reduce knowledge hiding by changing the layout of offices (e.g., demolishing physical walls; Singh, 2019) to maintain team stability.

Third, our results suggest that knowledge hiding mediates the association between TFL and TAL and NVT stability. On the one hand, CEOs could reduce knowledge hiding within their NVTs through TFL behaviors, such as involving their teams in the goal formation process during planning meetings and utilizing software to build internal communication channels (Burmeister et al., 2020). On the other hand, CEOs could devise a short-term incentive mechanism to reward team members for sharing knowledge to suppress knowledge hiding and improve NVT stability. For example, CEOs can clearly and formally compensate knowledge sharers by giving them stock ownership and economic rewards (Van Dijk et al., 2020).

Fourth, this study indicates that team collectivism strengthens the indirect effect of TFL on NVT stability through knowledge hiding. TFL behaviors may be operated alongside a high level of team collectivism to improve the effectiveness of leadership behaviors. On the one hand, CEOs could adopt more TFL behaviors to realize leadership functions when team collectivism is high. On the other hand, team collectivism stems from members’ experience of working alone or collaboratively (Wagner III et al., 2012). Thus, a transformational CEO could intervene in their members’ practice with managerial measures to intentionally cultivate team collectivism or select collectivist members to form the NVT. For example, the CEO could evidently and frequently involve other NVT members in decision-making, pursue collective goals and share responsibility, and plan informal social events for the team, such as dinners and travel activities (He et al., 2014).

### Limitations and future research

Several limitations should be noted and addressed in future research. First, the current study uses a three-wave longitudinal research design with data collected from multiple sources (i.e., CEOs and other members of NVTs) using a questionnaire. This method has its own advantages, but all of the study variables were not collected and computed at all periods. Thus, future researchers can use a full longitudinal research design in which all of the research model variables are measured in all periods. They can use an experimental design to validate this study’s findings. Second, considering that data were collected from a sample of 66 new ventures in China, the results may not apply to other countries and regions. Future research may be conducted in countries and regions with different cultural backgrounds to validate our theoretical model. The research would be useful for testing the cross-cultural generalizability of the results of this study. Third, in this study, we paid attention to general knowledge and did not distinguish between different types of knowledge (e.g., job knowledge, social knowledge, cultural or political knowledge, explicit knowledge, and tacit knowledge; Peng, 2013; Burmeister et al., 2020). Although the model explains that TFL and TAL reduce general knowledge hiding, and hiding general knowledge is harmful to NVT stability. Future studies would contribute more if they could specify the different types of knowledge. Last, this study only explores how TFL and TAL affect knowledge hiding and NVT stability. In the future, analyzing how other leadership behaviors, such as entrepreneurial leadership behaviors, which focus on leaders’ opportunity-oriented behaviors (Renko et al., 2015), affect knowledge hiding and NVT stability would provide interesting insights for NVT research.

## Conclusion

Based on social exchange theory and social information processing theory, this study proposes a framework to examine whether, how, and when TFL and TAL affect NVT stability. The results show that TFL and TAL are positively related to NVT stability and are negatively related to knowledge hiding, but TFL has effects on NVT stability and knowledge hiding beyond the effects of TAL. Knowledge hiding mediates the relationship between leadership behaviors and NVT stability. Furthermore, the study reveals an important finding—a high level of team collectivism corresponds to a stronger relationship between TFL and knowledge hiding and a greater indirect effect of TFL on NVT stability through knowledge hiding. Future research may use different research methods to verify our theoretical models in different cultural settings and distinguish among different types of knowledge. Future studies may also examine how other leadership behaviors that reflect NVT characteristics, such as entrepreneurial leadership behaviors, affect knowledge hiding and NVT stability.

## Data availability statement

The raw data supporting the conclusions of this article will be made available by the authors, without undue reservation.

## Author contributions

HM: conceptualization, funding acquisition, project administration, investigation, and writing—review and supervision. ST: investigation, validation, and writing—original draft. CZ: formal analysis, methodology, and writing—review and editing. All authors contributed to the article and approved the submitted version.

## Funding

This work was supported by the National Natural Science Foundation of China (Grant Number 71972084), the project of innovation team for the Jilin University (Grant Number 2022CXTD10), the Fundamental Research Funds for the Sichuan university (Grant No. 2021CXC23).

## Conflict of interest

The authors declare that the research was conducted in the absence of any commercial or financial relationships that could be construed as a potential conflict of interest.

## Publisher’s note

All claims expressed in this article are solely those of the authors and do not necessarily represent those of their affiliated organizations, or those of the publisher, the editors and the reviewers. Any product that may be evaluated in this article, or claim that may be made by its manufacturer, is not guaranteed or endorsed by the publisher.

## Supplementary material

The Supplementary material for this article can be found online at: https://www.frontiersin.org/articles/10.3389/fpsyg.2022.1001277/full#supplementary-material
