# Supplementary material for: CEOs’ leadership behaviors and new venture team stability: The effects of knowledge hiding and team collectivism
Source: Front Psychol. 2022 Nov 28;13:1001277. doi: 10.3389/fpsyg.2022.1001277 (PMC9745676; doi:10.3389/fpsyg.2022.1001277)
Supplement: Supplementary file 1 [file Table_1.doc]

# Appendix A. Questionnaire items and scales

| Variable | Items |
| --- | --- |
| Transformational leadership (TFL) | 1. On our team, the CEO is proud of them. (CH1) |
|  | 2. On our team, the CEO goes beyond self-interest for the good of the group. (CH2) |
|  | 3. On our team, the CEO has my respect. (CH3) |
|  | 4. On our team, the CEO displays power and confidence. (CH4) |
|  | 5. On our team, the CEO talks about his or her most important values and beliefs. (CH5) |
|  | 6. On our team, the CEO models an ethical standard. (CH6) |
|  | 7. On our team, the CEO considers the moral and ethical consequences of decisions. (CH7) |
|  | 8. On our team, the CEO emphasizes the collective mission. (CH8) |
|  | 9. On our team, the CEO talks optimistically about the future. (CH9) |
|  | 10. On our team, the CEO expresses confidence that goals will be achieved. (CH10) |
|  | 11. On our team, the CEO talks enthusiastically about what needs to be accomplished. (CH11) |
|  | 12. On our team, the CEO arouses awareness about important issues. (CH12) |
|  | 13. On our team, the CEO re-examines critical assumptions. (IS1) |
|  | 14. On our team, the CEO seeks differing perspectives when solving problems. (IS2) |
|  | 15. On our team, the CEO suggests new ways of looking at how to complete assignments. (IS3) |
|  | 16. On our team, the CEO gets others to look at problems from many different angles. (IS4) |
|  | 17. On our team, the CEO individualizes attention. (IC1) |
|  | 18. On our team, the CEO focuses on my strengths. (IC2) |
|  | 19. On our team, the CEO teaches and coaches other NVT members. (IC3) |
|  | 20. On our team, the CEO differentiates among us. (IC4) |
| Transactional leadership (TAL) | 1. On our team, the CEO clarifies what I can expect to receive when goals are achieved. (CR1) |
|  | 2. On our team, the CEO provides assistance in exchange for our efforts. (CR2) |
|  | 3. On our team, the CEO rewards my achievement. (CR3) |
|  | 4. On our team, the CEO recognizes my achievement. (CR4) |
|  | 5. On our team, the CEO focuses on my mistakes. (ME1) |
|  | 6. On our team, the CEO puts out fires. (ME2) |
|  | 7. On our team, the CEO tracks my mistakes. (ME3) |
|  | 8. On our team, the CEO concentrates on failures. (ME4) |
| Team collectivism | 1. The CEO is protective of and generous to loyal workers. (TC1) |
|  | 2. Decisions about changes in work methods are taken jointly by the CEO and other members of NVT. (TC2) |
|  | 3. Team members are taken care of like members of a family. (TC3) |
|  | 4. Everyone shares responsibility for the team’s failures as well as success. (TC4) |
|  | 5. Regardless of the hierarchical level, members take each other’s views into consideration. (TC5) |
|  | 6. Once someone is hired, the team takes care of that person’s overall welfare. (TC6) |
|  | 7. Everyone is kept informed about major decisions that affect the success of the team. (TC7) |
| Knowledge hiding | 1. When my teammates asked for some information, I pretended that I did not know the information. (PD1) |
|  | 2. When my teammates asked for some information, I said that I did not know, even though I did. (PD2) |
|  | 3. When my teammates asked for some information, I pretended I did not know what they were talking about. (PD3) |
|  | 4. When my teammates asked for some information, I said that I was not very knowledgeable about the topic. (PD4) |
|  | 5. When my teammates asked for some information, I agreed to help them but never really intended to. (EH1) |
|  | 6. When my teammates asked for some information, I agreed to help them but instead gave them information different from what they wanted. (EH2) |
|  | 7. When my teammates asked for some information, I told them that I would help them later but delayed as much as possible. (EH3) |
|  | 8. When my teammates asked for some information, I offered them some other information instead of what they really wanted. (EH4) |
|  | 9. When my teammates asked for some information, I explained that I would like to tell them but was not supposed to. (RH1) |
|  | 10. When my teammates asked for some information, I explained that the information was confidential and only available to colleagues on a particular project. (RH2) |
|  | 11. When my teammates asked for some information, I told them that my supervisor would not let anyone share this information or knowledge. (RH3) |
|  | 12. When my teammates asked for some information, I said that I would not answer their questions. (RH4) |
| New venture team stability | 1. NVT members (excluding the CEO) remained since joining the new venture. (ETS1) |
|  | 2. There were no changes in CEO from the beginning to the present. (ETS2) |
|  | 3. Team membership was stable; NVT members did not come and go since joining the new venture. (ETS3) |
